# Acaricidal Activity of Eugenol Based Compounds against Scabies Mites

**DOI:** 10.1371/journal.pone.0012079

**Published:** 2010-08-11

**Authors:** Cielo Pasay, Kate Mounsey, Graeme Stevenson, Rohan Davis, Larry Arlian, Marjorie Morgan, DiAnn Vyszenski-Moher, Kathy Andrews, James McCarthy

**Affiliations:** 1 Queensland Institute of Medical Research and Australian Centre for International and Tropical Health, University of Queensland, Brisbane, Queensland, Australia; 2 Eskitis Institute for Cell and Molecular Therapies, Griffith University, Nathan, Queensland, Australia; 3 Wright State University, Dayton, Ohio, United States of America; Direccion General de Epidemiologia, Peru

## Abstract

**Backgound:**

Human scabies is a debilitating skin disease caused by the “itch mite” *Sarcoptes scabiei*. Ordinary scabies is commonly treated with topical creams such as permethrin, while crusted scabies is treated with topical creams in combination with oral ivermectin. Recent reports of acaricide tolerance in scabies endemic communities in Northern Australia have prompted efforts to better understand resistance mechanisms and to identify potential new acaricides. In this study, we screened three essential oils and four pure compounds based on eugenol for acaricidal properties.

**Methodology/Principal Findings:**

Contact bioassays were performed using live permethrin-sensitive *S. scabiei* var *suis* mites harvested from pigs and permethrin-resistant *S. scabiei* var *canis* mites harvested from rabbits. Results of bioassays showed that clove oil was highly toxic against scabies mites. Nutmeg oil had moderate toxicity and ylang ylang oil was the least toxic. Eugenol, a major component of clove oil and its analogues –acetyleugenol and isoeugenol, demonstrated levels of toxicity comparable to benzyl benzoate, the positive control acaricide, killing mites within an hour of contact.

**Conclusions:**

The acaricidal properties demonstrated by eugenol and its analogues show promise as leads for future development of alternative topical acaricides to treat scabies.

## Introduction

Human scabies is caused by the “itch mite” *Sarcoptes scabiei* var *hominis*, a pathogen that burrows into skin and causes an inflammatory reaction that leads to pruritic lesions that may be followed by secondary bacterial infections. Serious complications may arise, especially following Group A streptococcal infection, leading to renal and heart disease [1]. Scabies infection causes significant morbidity, with an estimated 300 million people suffering from this infection at any one time [2].

Ordinary scabies can be treated with topical acaricides such as 5% permethrin. Crusted scabies, the more severe form of the disease, requires a combination of topical and oral treatment with ivermectin, the only orally administered drug available [3]. In Northern Australia where the disease is endemic, mass treatment with permethrin has been previously used as a control strategy [4]. While mass treatment programs can be effective [5] they can also provide an opportunity for selection and development of drug resistance in scabies mites. Using an *in vitro* acaricide assay we have shown an increased tolerance of scabies mites to permethrin [6] compared to 100% sensitivity prior to its widespread use [7]. Treatment failures have also been reported elsewhere to other topical acaricides such as benzyl benzoate [8], crotamiton [9] and lindane [10]. In 2004 *in vivo* and *in vitro* ivermectin resistance in Northern Australia was first reported, [11] and more recently, longitudinal evidence of increasing *in vitro* tolerance of scabies mites to ivermectin has been documented [12]. The possibility of widespread resistance of mites to current drugs has prompted recent efforts to better understand potential resistance mechanisms [13]–[17] and highlights an increasing need to identify new acaricidal agents.

Natural product extracts and compounds are potential sources of alternative acaricides [18]. Acaricidal activity of some plant essential oils and their chemical components has been demonstrated in several mite species such as poultry red mite, *Dermanyssus gallinae* [19], [20], rabbit mite, *Psoroptes cuniculi* [21], [22], honey bee mite, *Varroa destructor* [23], two-spotted spider mite, *Tetranychus urticae* [24], [25], [26], stored food mite, *Tyrophagus putrescentiae* [27], [28], house dust mite, *Dermatophagoides farinae* and *Dermatophagoides pteronyssinus* [29], [30], [31], human mite, *S. scabiei* var *hominis* [32], [33] and in sheep tick, *Ixodes ricinus* [34] and cattle tick, *Boophilus microplus* [35]. Insecticidal properties of some constituents of these plant oils have also been demonstrated in other arthropod species such as stored grain beetle, *Tribolium castaneum* [36], [37], maize weevil, *Sitophylus zeamais* [38], body louse, *Pediculus humanus corporis* [33], head louse, *Pediculus humanus capitis* [33], [39], [40] and mosquito vector, *Culex pipiens* [41] (Table S1).

**Figure 1:**
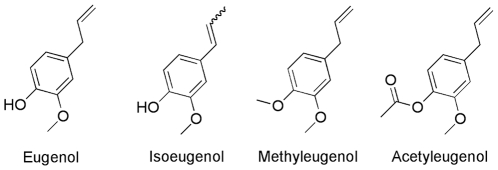
Chemical structures of compounds used in this study.

Of special interest amongst this list is an essential oil extracted from clove flower buds, *Eugenia caryophyllata*, a common food ingredient and a component of fragrances. Apart from being a traditional cure for diarrhea and other intestinal disorders in China, its active components have been demonstrated to have antimicrobial, insecticidal, antioxidant, antitumor and anaesthetic activity [42]. The primary component of clove oil is eugenol, which together with analogues, is a member of the phenylpropanoid class of chemical compounds (Fig. 1). Eugenol and several related analogues are minor components of other essential oils such as nutmeg, cinnamon and bay leaf oil.

To date, very limited screening of natural product extracts for acaricidal activity has been performed using *S. scabiei*. A major limitation in evaluating potential new acaricides has been the lack of a regular supply of adequate numbers of mites for reproducible *in vitro* testing. Previous studies have relied on harvesting *S. scabiei* var *hominis* mites from human crusted scabies patients [32] or testing candidate acaricides directly on scabies infected patients [33]. Such approaches are by necessity opportunistic in nature and raise ethical concerns. Neither of them allows for systematic or long term drug discovery approaches and severely limits biological investigations such as the study of resistance mechanisms.

In this study we used mites from two animal models of scabies, including a recently described porcine model [43], to systematically investigate the acaricidal activity of three essential oils and four pure compounds, all of which are derived from plants. The animal models and bioassays described here represent a significant improvement in our ability to evaluate potential new therapeutics for scabies.

## Materials and Methods

### Extracts and compounds

Clove oil and ylang ylang oil were purchased from Oil Garden Aromatheraphy (Fitzroy, VIC, Australia). Nutmeg Oil was purchased from Gumleaf Essentials (Lilydale, VIC, Australia).

Eugenol (Fluka Cat. No. 46100), isoeugenol (Aldrich Cat. No. 17206, mix of cis/trans, ∼1∶9), acetyleugenol (SAFC W24690-5-K), methyleugenol (Acros Cat. No. 259630050), mineral oil (M5904) and benzyl benzoate (M6630) were purchased from Sigma-Aldrich (St. Louis, MO). Stock concentrations of test compounds were prepared in mineral oil and freshly diluted to working concentrations (100 mM, 50 mM, 25 mM, 12.5 mM, 6.25 mM, 3.12 mM) immediately prior to use.

### Composition of essential oils

The chemical composition of essential oils and relative concentration of bioactive components may vary depending on a range of factors including the extraction method used, the geographical location where the plant was cultivated, time of harvest, storage method and part of the plant used. In this study, no chemical analysis to determine composition was performed on the three essential oils tested. However, reports from previous studies have shown that a representative sample of clove oil typically contains eugenol (69–89%), acetyleugenol (6–20%), beta-caryophyllene (1–10%), 2-heptanone (0.9%), ethyl hexanoate (0.7%), humulenol (0.3%), alpha humulene (0.2%), calacorene (0.1%) and calamenene (0.1%) [28], [42]. Jukic et al. reported that the major components of nutmeg oil are beta-pinene (23.9%), alpha-pinene (17.2%), myristicin (16.2%), terpinene-4-ol (7.9%), limonene (7.5%), gamma terpinene (6.8%) and the major components of the glycosidically bound volatile compounds are isoeugenol (46.1%) and methoxyeugenol (27.7%) [44]. The main constituents of ylang ylang oil previously obtained by different extraction methods (steam distillation, simultaneous distillation-solvent extraction and supercritical (CO_2_) extraction) were linalool (16.5–28.0%), germacrene-D (3.1–20.3%), benzyl benzoate (2.9–14.1%), benzyl acetate (6.2–17.0%), caryophyllene (2.9–3.9%) and *p*-methyl anisole (2.7–6.8%) [45]. Recently, a more specific and quantitative chemical composition analysis for distillation fraction II of Ylang Ylang oil reported methyl benzoate (34%), 4-methylanisole (19.8%) and benzyl benzoate (18.9%) as the major components [46].

### Scabies mites

Permethrin-sensitive *S. scabiei* var *suis* mites were collected from scabies infected pigs maintained at the Centre for Advanced Animal Science (CAAS), University of Queensland, Gatton Campus, Australia. To establish the colony, mites had been collected from naturally infected farm pigs in southeast Queensland with no known previous exposure to acaricide. An optimised immunosuppresion regimen was adapted to maintain mange indefinitely. Permethrin-resistant *S. scabiei* var *canis* mites were collected from colonies maintained on rabbits that had been under permethrin treatment for many years at Wright State University in Dayton, Ohio. Previous work with these mites has shown the median *in vitro* survival of sensitive mites in permethrin to be 4 hours, while in the same study median *in vitro* survival of resistant mites in permethrin was 15 hours. [17].

### Ethics statement

Approval for establishment and maintenance of the mite colony in pigs was obtained from the Animal Ethics committee of the Department of Employment, Economic Development and Innovation/University of Queensland (SA2009/07/294) and the Queensland Institute of Medical Research (QIMR-P630). Rabbits were maintained under a protocol approved by the Wright State University Laboratory Animal Care and Use Committee (A3632-01) in adherence to institutional guidelines for animal husbandry.

### Contact bioassays

Mite bioassays were conducted following a method previously described [17], with some modifications. Briefly, 35 µl of test compound diluted in mineral oil was spread evenly across the surface of a 30 mm×15 mm plastic petri dish (Proscitech, QLD, Australia) using a microtip. Live mites were placed directly into the petri dishes in direct contact with the test compounds and controls. Benzyl benzoate was used as the positive control acaricide while mineral oil alone was used as negative control. Mites were incubated in a 28°C humidified incubator and observed microscopically within the first 15–30 mins, hourly thereafter up to 6 hours, and again at the completion of the assay (24 hrs). Mortality was recorded by microscopic verification of absence of leg movement and gut peristalsis when touched with a probe. For the resistant mites, the bioassays undertaken to test the three essential oils (clove, nutmeg, ylang ylang) and the 4 compounds (eugenol, isoeugenol, acetyleugenol and methyleugenol) were each performed in duplicate with 10 mites/concentration of oil or compound. Hence, the total sample size for each oil or compound tested is n = 20 resistant mites/concentration. For sensitive mites, the bioassays were performed in the same manner. However, these bioassays were performed twice so total sample size or n = 40 sensitive mites/concentration of oil or compound tested. Duplicate assays were performed independently to control for intra-observer bias.

### Data analysis and statistics

Median survival times of scabies mites in essential oils and test compounds were determined by Kaplan Meier survival analysis and significant differences between survival curves were calculated by Log-rank tests using the GraphPad Prism software package, version 5 (La Jolla, CA,USA). Effective concentration (EC_50_) values were determined by dose-response analysis and best fit curves generated by nonlinear regression analysis also in GraphPad Prism. Significant differences between EC_50_ values between compounds tested were determined using Student's t tests. To determine which doses of compound produce significant differences in toxicity between sensitive and resistant mites, Fisher's exact test on a single sided hypothesis test was performed in SPSS (Chatswood, NSW, Australia).

## Results

### Acaricidal activities of essential oils

**Figure 2:**
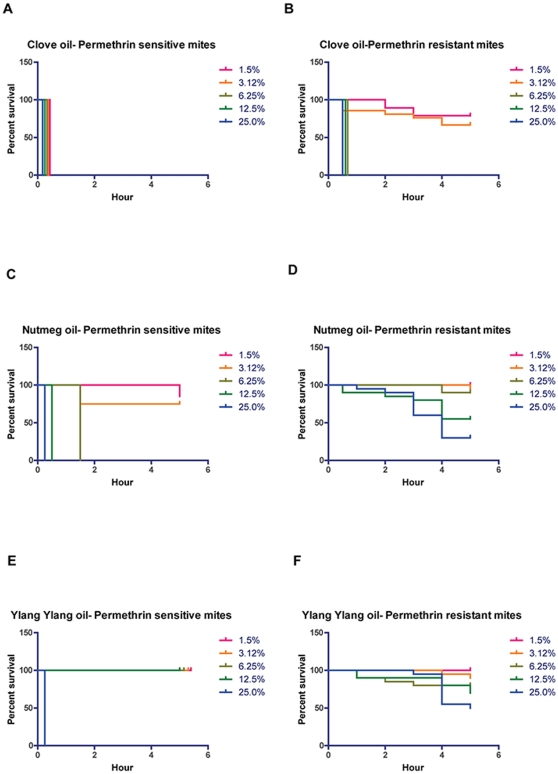
Survival of permethrin-sensitive and permethrin-resistant mites in different essential oils in contact bioassays. Scabies mites were exposed to two-fold dilution of the essential oils starting at 25%. N = 40 sensitive mites/concentration; N = 20 resistant mites/concentration. **A.** Survival of sensitive mites in different concentrations of clove oil. **B.** Survival of resistant mites in different concentrations of clove oil. **C.** Survival of sensitive mites in different concentrations of nutmeg oil. **D.** Survival of resistant mites in different concentrations of nutmeg oil. **E.** Survival of sensitive mites in different concentrations of ylang ylang oil. **F.** Survival of resistant mites in different concentrations of ylang ylang oil.

The median survival times of permethrin-sensitive and resistant scabies mite populations exposed to clove oil, nutmeg oil and ylang ylang oil are shown in Table 1. At all concentrations tested (1.56%–25%), contact with clove oil resulted in 100% mortality of permethrin-sensitive mites after 0.25 hours. Permethrin-resistant mites died at the same time but required higher concentrations (≥6.25%) of clove oil (Table 1 and Fig. 2A–B). A dose dependent change in median survival time of permethrin-sensitive mites was observed when mites were exposed to nutmeg oil, with a very low mortality observed at concentrations below 6.25%. In contrast, permethrin-resistant mites were less susceptible to nutmeg oil with median mortality occurring only at 25% oil after 4 hours (Table 1 and Fig. 2C–D). Permethrin-sensitive and permethrin-resistant mites were both tolerant to ylang ylang oil, with significant mortality only occurring at the highest concentration tested. Of note, however, the acaricidal effect was faster in sensitive mites than in resistant mites (Table 1 and Fig. 2E–F).

**Table 1:** Median survival time of scabies mites in different essential oils in contact bioassays.

			Median survival time (hour)			
Concentration	Clove oil		Nutmeg oil		YlangYlang oil	
(%)	Sensitive mites	Resistant mites	Sensitive mites	Resistant mites	Sensitive mites	Resistant mites
25	0.25	0.25	0.25	4	1	5
12.5	0.25	0.25	0.5	na	na	na
6.25	0.25	0.25	1.5	na	na	na
3.12	0.25	na	na	na	na	na
1.56	0.25	na	na	na	na	na

N = 40 sensitive mites/concentration; N = 20 resistant mites/concentration; na  =  no acaricidal activity.

### Acaricidal activity of pure compounds

**Figure 3:**
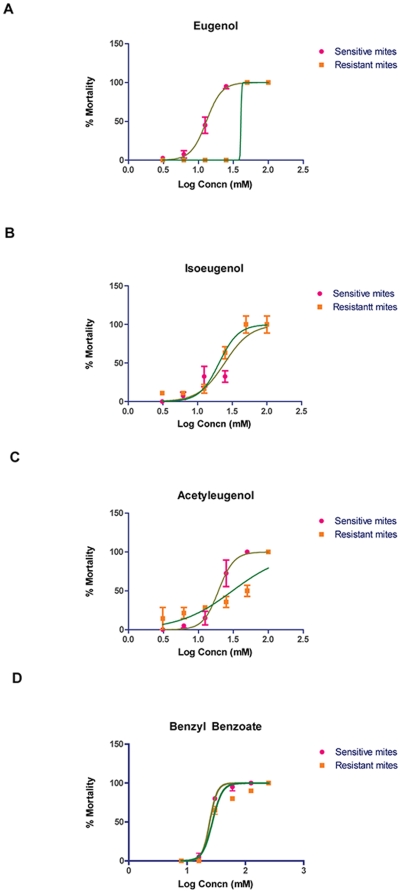
Dose-response curves: eugenol and its analogues against scabies mites. Percent mortality of scabies mites plotted against log of concentration of compounds tested compared to benzyl benzoate (positive control acaricide); N = 40 sensitive mites/concentration; N = 20 resistant mites/concentration. **A.** Mortality of sensitive and resistant mites in eugenol. **B.** Mortality of sensitive and resistant mites in isoeugenol. **C.** Mortality of sensitive and resistant mites in acetyleugenol. **D.** Mortality of sensitive and resistant mites in benzyl benzoate.

The acaricidal activities of eugenol, a major component of clove oil, and its minor component acetyleugenol as well as the related analogues isoeugenol and methyleugenol, were tested in contact bioassays against the two scabies mite populations. Mortality in the two mite populations at different concentrations of the compound were plotted as dose-response curves (Fig. 3A–D) and EC_50_ values were derived after an hour of observation (Table 2). For both mite populations, there was no significant difference between the activity observed for the positive control acaricide (benzyl benzoate) and the test compounds eugenol, acetyleugenol, and isoeugenol (p>0.11). In contrast, methyleugenol had no acaricidal effect in sensitive mites after the first hour of observation. This compound only displayed activity in resistant mites after 24 hours at the highest concentration tested (100 mM).

**Table 2:** Acaricidal activity of eugenol and its analogues against permethrin-sensitive and resistant mites in contact bioassays.

Compound	Sensitive mitesEC_50_ (mM)	Resistant mitesEC_50_ (mM)
Eugenol	13.0(11.9–14.3)**	40.7#
Isoeugenol	24.6 (19.4–31.2)	32.1 (25.7–40.2)
Acetyleugenol	19.4 (16.5–22.8)	30.8 (19.1–49.6)
Benzyl Benzoate*	24.5 (22.9–26.2)	27.2 (23.7–31.25)

N = 40 sensitive mites/concentration; N = 20 resistant mites/concentration; EC_50_ values calculated after 1 hour of observation;

*Positive control acaricide;

**95% confidence interval (CI);

# no CI.

**Figure 4:**
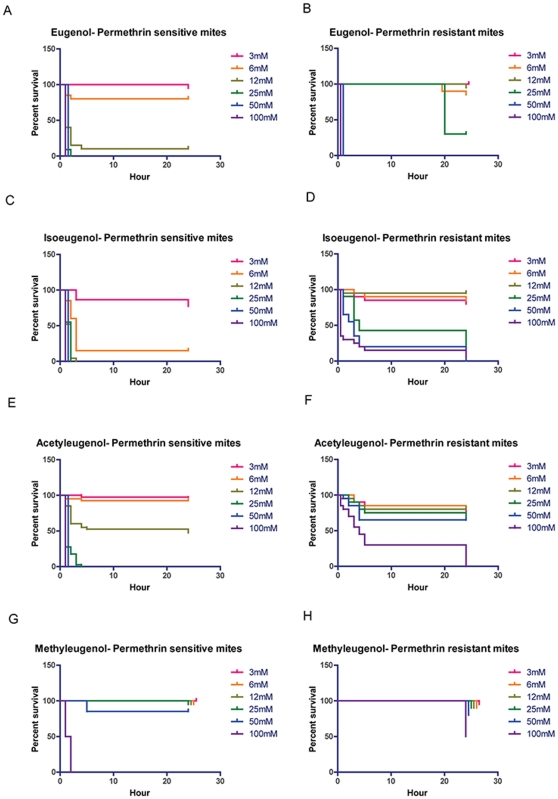
Survival of permethrin-sensitive and resistant mites in eugenol and analogues in contact bioassays. Scabies mites were exposed to two-fold dilution of the compounds starting at 100 mM. N = 40 sensitive mites/concentration; N = 20 resistant mites/concentration. **A.** Survival of sensitive mites in different concentrations of eugenol. **B.** Survival of resistant mites in different concentrations of eugenol. **C.** Survival of sensitive mites in different concentrations of isoeugenol. **D.** Survival of resistant mites in different concentrations of isoeugenol. **E.** Survival of sensitive mites in different concentrations of acetyleugenol. **F.** Survival of resistant mites in different concentrations of acetyleugenol. **G.** Survival of sensitive mites in different concentrations of methyleugenol. **H.** Survival of resistant mites in different concentrations of methyleugenol.

At concentrations ≥12 mM, median survival time of sensitive mites in eugenol was not affected by the concentration of the compound (Fig. 4A–B and Table 3), while a dose dependent effect on median survival time was observed in sensitive and resistant mites exposed to isoeugenol (Fig. 4C–D and Table 3). There was no acaricidal activity at 24 hours at isoeugenol doses <6 mM and <25 mM for sensitive and resistant mites, respectively. Acetyleugenol was toxic to resistant mites only at a high concentration (Fig. 4E–F and Table 3) with no acaricidal effect observed below 100 mM. The same was observed with sensitive and resistant mites exposed to methyleugenol (Fig. 4G–H and Table 3).

**Table 3:** Median survival time of scabies mites in different compounds in contact bioassays.

			Median survival time (hour)					
	Eugenol		Isoeugenol		Acetyleugenol		Methyleugenol	
Concn (mM)	Sensitive mites	Resistant mites	Sensitive mites	Resistant mites	Sensitive mites	Resistant Mites	Sensitive mites	Resistant mites
100	1	1	1	1	1	4	1	24
50	1	1	1	3	1	na	na	na
25	1	24	2	4	1	na	na	na
12	1	na	2	na	24	na	na	na
6	na	na	3	na	na	na	na	na
3	na	na	na	na	na	na	na	na

N = 40 sensitive mites/concentration; N = 20 resistant mites/concentration; na  =  no acaricidal activity.

To determine which concentration of each compound produced discriminatory differences in mortality between permethrin-sensitive and permethrin-resistant mites, the proportion of deaths at each concentration were compared by Fisher's exact test. There was a significant difference in mortalities between the two mite populations following exposure to eugenol at levels between 12.5 mM and 25 mM; in acetyleugenol, between 25 mM and 100 mM; in isoeugenol between 50 mM and 100 mM and in methyleugenol at 100 mM (Table 4). These results show possible cross- resistance to permethrin of compounds tested.

**Table 4:** Toxicity of eugenol and analogues against permethrin-sensitive and resistant mites in contact bioassays.

Compound	Concentration (mM)	Sensitive mites (% mortality)	Resistant mites (% mortality)	p-value (single sided Fisher's exact test)
Eugenol	100502512.5	1001009545	10010000	nsns<0.00010.0001
Isoeugenol	100502512.5	10010032.532.5	7565195	0.00250.0002nsns
Acetyleugenol	100502512.5	10010072.515	70352520	0.0008<0.00010.0006ns
Methyleugenol	100502512.5	1002000	50201010	<0.0001nsnsns

N = 40 sensitive mites/concentration; N = 20 resistant mites/concentration; % mortality after 1 hour of observation; ns  =  p value>0.0025.

## Discussion

In this study, *in vitro* bioassays were conducted using mites from two models to screen essential oils and pure compounds for acaricidal activity. The differences observed in the mite killing properties of clove, nutmeg and ylang ylang oils are attributable to variation in their chemical compositions as reported in previous studies. The levels of toxicity of the pure active compounds originating from the essential oils were equivalent or higher than benzyl benzoate, the positive control acaricide used in this study, and reflected in the decreased median survival time of scabies mites in contact bioassays. These data further validate the acaricidal properties of eugenol and the related analogues isoeugenol and acetyleugenol against scabies mites. A notable finding of this study, however, is the absence of acaricidal effect of methyleugenol at lower concentrations (below 100 mM) against scabies mites. This is in contrast to results obtained in house dust mites, *D. farinae* and *D. pteronyssinus*, where it is more toxic than eugenol, isoeugenol and acetyleugenol [29]. In house dust mites it was observed that methyleugenol caused a loss of glossiness of the mite's cuticle resulting in dessication, leading to death. While this mode of action of methyleugenol may be the same in scabies mites the compounds tested in this study were diluted in mineral oil making it difficult to make direct comparisons.

The variability observed in the toxicity of the phenylpropanoids tested against scabies mites in this study appears dependent on their individual structures. The acaricidal properties of eugenol and isoeugenol were different suggesting that the position of the double bond in the C_6_-C_3_ system is important in activity. The position of the double bond in this system has been shown to affect aphid biology by interfering with mitochondrial respiration and other target sites [47].

When considering the therapeutic potential of new acaricides, it is important to select compounds and concentrations demonstrating minimal cross resistance with existing acaricides. Therefore, two mite colonies were compared. The porcine mite colony has never been exposed to permethrin, or any other acaricide. In contrast, the second colony of mites from infected rabbits was maintained under permethrin pressure for many years and are resistant to permethrin *in vitro* [17]. It is possible that the significant differences in toxicity between sensitive and resistant mites in various doses of the eugenol-derived compounds observed may indicate some degree of cross resistance with permethrin. However, as it was not possible to perform repeat experiments with the resistant mites, these results should be interpreted with some caution. Higher levels of glutathione-S-transferase (GST) enzymes and upregulation of GST transcription in resistant mites compared to sensitive mites has been detected, and indicative of a metabolic basis for permethrin resistance in this mite colony [48]. Whether a similar pattern of resistance observed in these studies with the compounds tested is mediated by the same metabolic detoxification by GSTs as previously observed in permethrin resistant mites will need further investigation.

The acaricidal properties demonstrated by clove oil and its major component, eugenol and minor component isoeugenol and analogue acetyleugenol against scabies mites in this study paves the way for developing new alternative topical acaricide to treat scabies. In addition to robust bioassays that facilitate scabies drug discovery, other factors such as cost, availability and tolerance to application should also be addressed. For example, while benzyl benzoate is highly effective *in vitro*, it can cause significant irritation when used to treat people and needs to be diluted for use in children. Therapeutic use of eugenol-based compounds would therefore require further safety and *in vivo* efficacy before clinical use.

## Supporting Information

Table S1 List of essential oils of botanical origin with known acaricidal and insecticidal properties.
(0.06 MB DOC)

## References

[1] Currie BJ, Carapetis JR (2000). Skin infections and infestations in Aboriginal communities in northern Australia.. Australas J Dermatol.

[2] Taplin D, Meinking TL, Chen JA, Sanchez R (1990). Comparison of crotamiton 10% cream (Eurax) and permethrin 5% cream (Elimite) for the treatment of scabies in children.. Pediatric Dermatol.

[3] Currie BJ, McCarthy JS (2010). Permethrin and ivermectin for scabies.. N Engl J Med.

[4] Carapetis JR, Connors C, Yarmirr D, Krause V, Currie BJ (1997). Success of a scabies control program in an Australian aboriginal community.. Pediatric Infect Dis J.

[5] Taplin D, Porcelain SL, Meinking TL, Athey RL, Chen JA (1991). Community control of scabies: a model based on use of permethrin cream.. Lancet.

[6] Walton SF, Myerscough MR, Currie BJ (2000). Studies *in vitro* on the relative efficacy of current acaricides for *Sarcoptes scabiei* var *hominis*.. Trans R Soc Trop Med Hyg.

[7] Fraser J (1994). Permethrin: a top end viewpoint and experience.. Med J Aust.

[8] Hernandez-Perez E (1983). Resistance to antiscabietic drugs.. J Am Acad Dermatol.

[9] Coskey RJ (1979). Scabies-resistance to treatment with crotamiton.. Arch Dermatol.

[10] Roth W (1991). Scabies resistance to lindane 1% lotion and crotamiton 10% cream.. J Am Acad Dermatol.

[11] Currie BJ, Harumal P, McKinnon M, Walton SF (2004). First documentation of *in vivo* and *in vitro* ivermectin resistance in *Sarcoptes scabiei*.. Clin Infect Dis.

[12] Mounsey KE, Holt DC, McCarthy JS, Currie BJ, Walton SF (2009). Longitudinal evidence of increasing *in vitro* tolerance of scabies mites to ivermectin in scabies-endemic communities.. Arch Dermatol.

[13] Mounsey KE, Dent JA, Holt DC, McCarthy JS, Currie BJ (2007). Molecular characterisation of a pH-gated chloride channel from *Sarcoptes scabiei*.. Invert Neurosci.

[14] Mounsey KE, Holt DC, McCarthy JS, Currie BJ, Walton SF (2008). Scabies: molecular perspectives and therapeutic implications in the face of emerging drug resistance.. Future Microbiol.

[15] Pasay C, Walton S, Fischer K, Holt D, McCarthy J (2006). PCR-based assay to survey for knockdown resistance to pyrethroid acaricide in human scabies mites (*Sarcoptes scabiei* var *hominis*).. Am J Trop Med Hyg.

[16] Pasay C, Arlian L, Morgan M, Vyszenski-Moher D, Rose A (2008). High-resolution melt analysis for the detection of a mutation associated with permethrin resistance in a population of scabies mites.. Med Vet Entomol.

[17] Pasay C, Arlian L, Morgan M, Gunning R, Rossiter L (2009). The effect of insecticide synergists on the response of scabies mites to pyrethroid acaricides.. PLoS Negl Trop Dis.

[18] Williamson EM (2007). The medicinal use of essential oils and their components for treating lice and mite infestations. Natural Product Communications..

[19] George DR, Masic D, Sparagano OAE, Guy J (2009). Variation in chemical composition and acaricidal activity against *Dermanyssus gallinae* of four eucalyptus essential oils.. Exp Appl Acarol.

[20] George DR, Sparagano OAE, Port G, Okello E, Shiel RS (2010). Environmental interactions with the toxicity of plant essential oils to the poultry red mite *Dermanyssus gallinae*.. Med Vet Entomol.

[21] Perrucci S, Cioni PL, Flamini G, Morelli I, Macchioni G (1994). Acaricidal agents of natural origin against *Psoroptes cuniculi*.. Parassitologia.

[22] Fichi G, Flamini G, Giovanelli F, Otranto D, Perrucci S (2007). Efficacy of an essential oil of *Eugenia caryophyllata* against *Psoroptes cuniculi*.. Exp Parasitol.

[23] Damiani N, Gende LB, Bailac P, Marcangeli JA, Eguaras MJ (2009). Acaricidal and insecticidal activity of essential oils on *Varroa destructor* (Acari: Varroidae) and *Apis mellifera* (Hymenoptera:Apidae).. Parasitol Res.

[24] Miresmailli S, Bradbury R, Isman MB (2006). Comparative toxicity of *Rosmarinus officinalis* L. essential oil and blends of its major constituents against *Tetranychus urticae* Koch (Acari: Tetranychidae) on two different host plants.. Pest Manag Sci.

[25] Araujo CP, da Camara CA, Neves IA, Ribeiro Nde C, Gomes CA (2010). Acaricidal activity against *Tetranychus urticae* and chemical composition of peel essential oils of three Citrus species cultivated in NE Brazil.. Nat Prod Commun.

[26] Cavalcanti SC, Niculau Edos S, Blank AF, Camara CA, Araujo IN (2010). Composition and acaricidal activity of *Lippia sidoides* essential oil against two-spotted spider mite (*Tetranychus urticae* Koch).. Bioresour Technol.

[27] Macchioni F, Cioni PL, Flamini G, Morelli I, Perrucci S (2002). Acaricidal activity of pine essential oils and their main components against *Tyrophagus putrescentiae*, a stored food mite.. J Agric Food Chem.

[28] Kim EH, Kim HK, Choi DH, Ahn YJ (2003). Acaricidal activity of clove bud oil compounds against *Tyrophagus putrescentiae* (Acari: Acaridae).. Appl Entomol Zool.

[29] Kim EH, Kim HK, Ahn YJ (2003). Acaricidal activity of clove bud oil compounds against *Dermatophagoides farinae* and *Dermatophagoides pteronyssinus* (Acari: Pyroglyphidae).. J Agr Food Chem.

[30] Kim HK, Yun Yk, Ahn YJ (2008). Fumigant toxicity of cassia bark and cassia ans cinnamon oil compounds to *Dermatophagoides farinae* and *Dermatophagoides pteronyssinus* (Acari: Pyroglyphidae).. Exp Appl Acarol.

[31] Jeong EY, Kim MG, Lee HS (2009). Acaricidal activity of triketone analogues derived from *Leptospermum scoparium* oil against house-dust and stored-food mites.. Pest Manag Sci.

[32] Walton SF, McKinnon M, Pizutto S, Dougall A, Williams E (2004). Acaricidal activity of *Melaleuca alternifolia* (tea tree) oil: *in vitro* sensitivity of *Sarcoptes scabiei* var *hominis* to terpinene-4-ol.. Arch Dermatol.

[33] Oladimeji FA, Orafidiya OO, Ogunniyi TAB, Adewunmi TA (2000). Pediculocidal and scabicidal properties of *Lippia multiflora* essential oil.. J Ethnopharmacol.

[34] Elmhalli FH, Palsson K, Orberg J, Jaenson TG (2009). Acaricidal effects of Corymbia citriodora oil containing para-menthane-3,8-diol against nymphs of *Ixodes ricinus* (Acari:Ixodidae).. Exp Appl Acarol.

[35] Ribeiro VL, Dos Santos JC, Bordignon SA, Apel MA, Henriques AT (2010). Acaricidal properties of volatile extracts from *Hesperozygis ringens* (Lamiaceae) on the cattle tick *Riphicephalus* (*Boophilus) microplus*.. Bioresour Technol.

[36] Ho SH, Cheng LPL, Sim KY, Tan HTW (1994). Potential of cloves (*Syzygium aromaticum* (L.) Merr. and Perry as a grain protectant against *Tribolium castaneum* (Herbst) and *Sitophilus zeamais* Motsch.. Postharvest Biology and Technology.

[37] Olivero-Verbel J, Nerio LS, Stashenko EE (2010). Bioactivity against *Tribolium castaneum* Herbst (Coleoptera: Tenebrionidae) of *Cymbopogon citratus* and *Eucalyptus citriodora* essential oils grown in Colombia.. Pest Manag Sci.

[38] Liu ZL, Chu SS, Liu QR (2010). Chemical composition and insecticidal activity against *Sitophilus zeamais* of the essential oils of *Artemisia capillaries* and *Artemisia mongolica.*. Molecules.

[39] Yang YC, Lee SH, Lee WJ, Choi DH, Ahn YJ (2003). Ovicidal and adulticidal effects of *Eugenia caryophyllata* bud and leaf oil compounds on *Pediculus capitis*.. J Agric Food Chem.

[40] Choi HY, Yang YC, Lee SH, Clark JM, Ahn YJ (2010). Efficacy of spray formulations containing binary mixtures of clove and eucalyptus oils against susceptible and pyrethroid/malathion-resistant head lice (Anoplura:Pediculidae).. J Med Entomol.

[41] Koliopoulus G, Pitarokili D, Kioulos E, Michaelakis A, Tzakou O Chemical composition and larvicidal evaluation of Mentha, Salvia and Melissa essential oils against West Nile virus mosquito *Culex pipiens*.. Parasitol Res Apr 20 (Epub ahead of print).

[42] Chaieb K, Hajlaoui H, Zmantar T, Kahla-Nakbi AB, Rouabhia M (2007). The chemical composition and biological activity of clove essential oil, *Eugenia caryophyllata* (*Syzigium aromaticum* L. Myrtaceae): a short review.. Phytother Res.

[43] Mounsey K, Ho M, Kelly A, Willis C, Pasay C (2010). A tractable experimental model for study of human and animal scabies.. PLoS Negl Trop Dis.

[44] Jukic M, Politeo O, Milos M (2006). Chemical composition and antioxidant effect of free volatile aglycones from nutmeg (*Myristica fragrans* Houtt.) compared to its essential oil.. Croatia Chemica Acta.

[45] Stashenko EE, Prada NQ, Martinez JR (1996). HRGC/FID/NPD and HRGC/MSD Study of Colombian Ylang-Ylang (*Cananga odorata*) oils obtained by different extraction techniques.. J High Resol Chromatogr.

[46] Hongratanaworakit T, Buchbauer G (2004). Evaluation of the harmonizing effect of ylang ylang oil on humans after inhalation.. Planta Medica.

[47] Tewary DK, Bhardwaj A, Sharma A, Sinha AK, Shanker A (2006). Bioactivity and structure-activity relationship of natural methoxylated phenylpropenes and their derivative against *Aphis craccivora* Koch (Hemiptera:Aphididae).. J Pest Sci.

[48] Mounsey KE, Pasay CJ, Arlian LG, Morgan MS, Holt DC (2010). Increased transcription of Glutathione S-transferases in acaricide exposed mites.. Parasit Vectors.

