# Supplementary material for: Acaricidal Activity of Eugenol Based Compounds against Scabies Mites
Source: PLoS One. 2010 Aug 11;5(8):e12079. doi: 10.1371/journal.pone.0012079 (PMC2920318; doi:10.1371/journal.pone.0012079)
Supplement: Table S1 — List of essential oils of botanical origin with known acaricidal and insecticidal properties. (0.06 MB DOC) [file pone.0012079.s001.doc]

**Table1. List of essential oils of botanical origin with known acaricidal and insecticidal properties**

| **Essential Oil** | **Plant species origin** | **Major component identified or tested** | **Mite/other arthropod species tested** | **Reference** |
| --- | --- | --- | --- | --- |
| Eucalyptus | *Eucalyptus citriodora*  *E. staigeriana*  *E.globulus*  *E.radiata* | 6-octenal  2,6-octadien-1-ol  bicyclohex-2-ene  1,8 cineole  1,8 cineole | *Dermanyssus gallinae* (poultry red mite) | [19] |
| Manuka  Cade  Pennyroyal  Thyme  Garlic  Clove bud oil  Cinnamon bark  (most acaricidal out of 50 tested) | *Leptospermum scoparium*  *Juniperus oxycedrus*  *Mentha pulegium*  *Thymus vulgaris*  *Allium sativum*  *Eugenia caryophyllata*  *Cinnamomum. zeylanicum* | *components not tested | *D. gallinae* | [20] |
| Lavender | *Lavandula angustifolia* | linalool | *Psoroptes cuniculi*  (rabbit mite) | [21] |
| Eugenia | *E. caryophyllata* | *Components not tested | *P. cuniculi* | [22] |
| Lavender  Lavendin  Laurel  Thyme | *L. officinalis*  *L hybrida*  *Laurus nobilis*  *Thymus vulgaris* | linalool  1,8 cineole  thymol | *Varroa desructor*  (honeybee mite)  *Apis mellifera*  (honey bee) | [23] |
| Rosemary | *Rosmarinus officinalis* | 1,8 cineole  camphor  alpa-pinene | *Tetranychus urticae* (two-spotted spider mite) | [24] |
| Citrus peel | *Citrus sinensis* var *pena*  *C. senensis* var  *mimo*  *C. aurantium* | limonene  alpha-pinene  myrcene  linalool  eugenol  phosphine | *T. urticae* | [25] |
| Lippia | *Lippia sidoides* | thymol  carvacrol | *T.urticae* | [26] |
| Pine | *Pinus pinea* | 1,8 cineole,  limonene | *Tyrophagus putrescentiae* (stored food mite) | [27] |

Table1 (continued)

| **Essential Oil** | **Plant species origin** | **Major component identified or tested** | **Mite/other arthropod species tested** | **Reference** |
| --- | --- | --- | --- | --- |
| Clove bud | *E. caryophyllata* | acetyleugenol  beta-caryophyllene  eugenol  alpha-humulene  isoeugenol  methyleugenol | *T. putrescentiae* | [28] |
| Clove bud | *E. caryophyllata* | eugenol, acetyleugenol isoeugenol, methyl eugenol | *Dermatophagoides farinae, D.pteronyssinus* (house dust mite) | [29] |
| Cassia bark  Cassia  Cinnamon | *Cinnamomum zeylanicum* | E-cinnamaldehyde  salicylaldehyde  benzaldehyde  menthol, thymol  alpha terpineol | *D. farinae*  *D. pteronyssinus* | [30] |
| Leptospermum | *Leptospermum scoparium* | 6-isovaleryl-2,2,4,4-tetramethyl-1,3,5-cyclohexanetrione  2,2,4,4,6,6-hexamethyl-1,3,5-  cyclohexanetrione | *D. farinae*  *D. pteronyssinus*  *T. putrescentiae* | [31] |
| Tea tree | *Malaleuca alternifolia* | terpinen-4-ol  alpha-terpineol  1,8-cineole | *Sarcoptes scabiei*  var *hominis* (human mite) | [32] |
| Lippia | *Lippia multiflora* | *components not tested | *S. scabiei*  var *hominis* (human mite)  *P. humanus corporis (body louse)*  *P. humanus capitis* (head louse) | [33] |
| Eucalyptus | *Corymbia citriodora* | *para-*menthane-3,8-diol | *Ixodes ricinus* (tick) | [34] |
| Hesperozygis | *Hesperozygis ringens* | pulegone | *Boophilus microplus* (cattle tick) | [35] |
| Clove | *Syzygium aromaticum* | *components not tested | *Tribolium castaneum* (stored grain beetle)  *Sitophylus zeamais* (maize weevil) | [36] |
| Cymbopogon  Eucalyptus | *Cymbopogon citratus*  *E. citriadora* | geranial  neral  geraniol, citronellal  isopulegol,citronellol | *T. castaneum* | [37] |

|  |  |  |  |  |
| --- | --- | --- | --- | --- |
| **Table1 (continued)** | | | | |
| **Essential Oil** | **Plant species origin** | **Major component identified or tested** | **Mite/other arthropod species tested** | **Reference** |
| Artemisia | *Artemisia capillaris*  *A.mongolica* | 1, 8 cineole  germacrene D  camphor  alpha-pinene  germacrene D  gamma terpinene  (above components derived but not tested) | *S. zeamais* | [38] |
| Clove bud | *E. caryophyllata* | eugenol, isoeugenol, methyl eugenol | *P. capitis* (head louse) | [39] |
| Clove  Eucalyptus | *E. caryophyllata*  *E. globulus* | *Components not tested; binary mixtures tested | *P. humanus capitis* | [40] |
| Mentha  Melissa  Salvia | *Mentha spicata*  *M. longifolia*  *M. suaveolens*  *Melissa officinalis*  *Salvia fruticosa*  *S.pomifera(calycina)*  *S.pomifera(pomifera)* | piperitenone oxide  1,8 cineole | *Culex pipiens* | [41] |
|  |  |  |  |  |
